# Supplementary material for: Multiomics Analysis of a DNAH5-Mutated PCD Organoid Model Revealed the Key Role of the TGF-β/BMP and Notch Pathways in Epithelial Differentiation and the Immune Response in DNAH5-Mutated Patients
Source: Cells. 2022 Dec 12;11(24):4013. doi: 10.3390/cells11244013 (PMC9776854; doi:10.3390/cells11244013)
Supplement: Supplementary file 1 [file cells-11-04013-s001.zip › Supplementary figure legend.pdf]

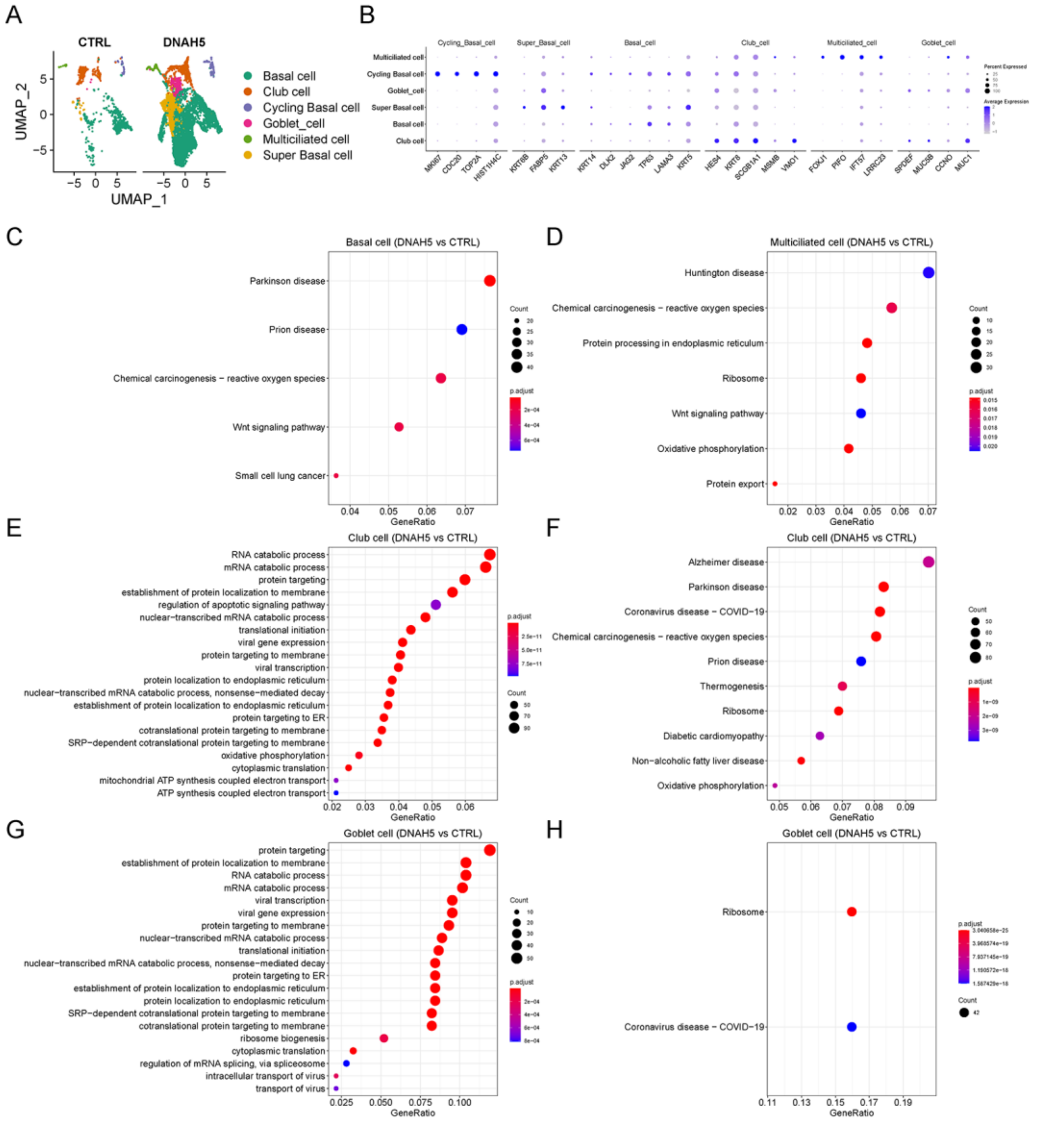


**Figure S1. (A)** UMAP plots of expression from scRNA-seq of child airway organoids (*DNAH5*-mutated and control). **(B)** Dot plot showing the relative expression and the percentage of cells expressing selected marker genes across scRNA-seq clusters. **(C)** The KEGG enrichment of basal cells in the two groups showing the differential expression. **(D)** The KEGG enrichment of multiciliated cells in the two groups showing the differential expression. **(E-F)** GO enrichment analysis of biological processes and KEGG enrichment in club cells (DNAH5 vs. CTRL) showing the differential expression of immune-related genes. **(G-H)** GO enrichment analysis of biological processes and KEGG enrichment in goblet cells (DNAH5 vs. CTRL).

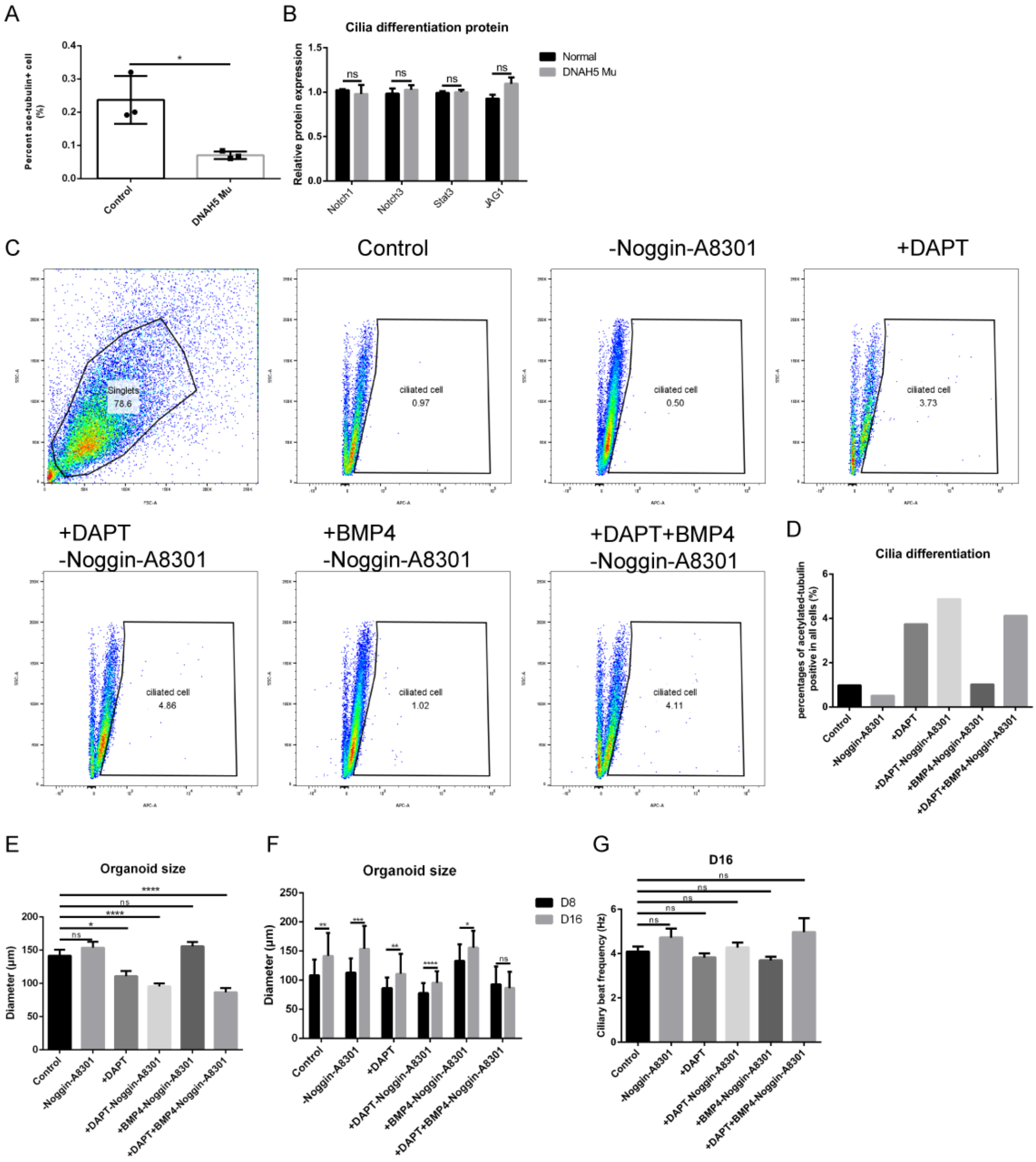


**Figure S2. (A)**The percentage of ace-tubulin+ cells in Hoechst33342+ cells. **(B)** The relative protein expression related to cilia differentiation in proteomic analysis. **(C)** FACS plots show that single acetylated-α-tubulin-positive cells were identified in child airway organoids induced by different treatments for 8 days. **(D)** Bar plots depicting the percentages of acetylated tubulin-positive cells in child airway organoids differentiated in treatments for 8 days. The highest percentage of ciliated cells was observed in the +DAPT-Noggin-A8301 group. **(E)** The organoid size was measured at D16. **$p<0.01$, ***$p<0.001$, ns: no significance; n=20 per group. **(F)** The organoids at D16 were larger than those at D8 in most groups. *$p<0.05$, **$p<0.01$, ***$p<0.001$, ns: no significance; n=20 per group. **(G)** The ciliary beat frequency of normal child airway organoids was recorded at D16. ns: no significance; n=20 per group.
