# Supplementary figures and images for: Multiomics Analysis of a DNAH5-Mutated PCD Organoid Model Revealed the Key Role of the TGF-β/BMP and Notch Pathways in Epithelial Differentiation and the Immune Response in DNAH5-Mutated Patients
Source: Cells. 2022 Dec 12;11(24):4013. doi: 10.3390/cells11244013 (PMC9776854; doi:10.3390/cells11244013)

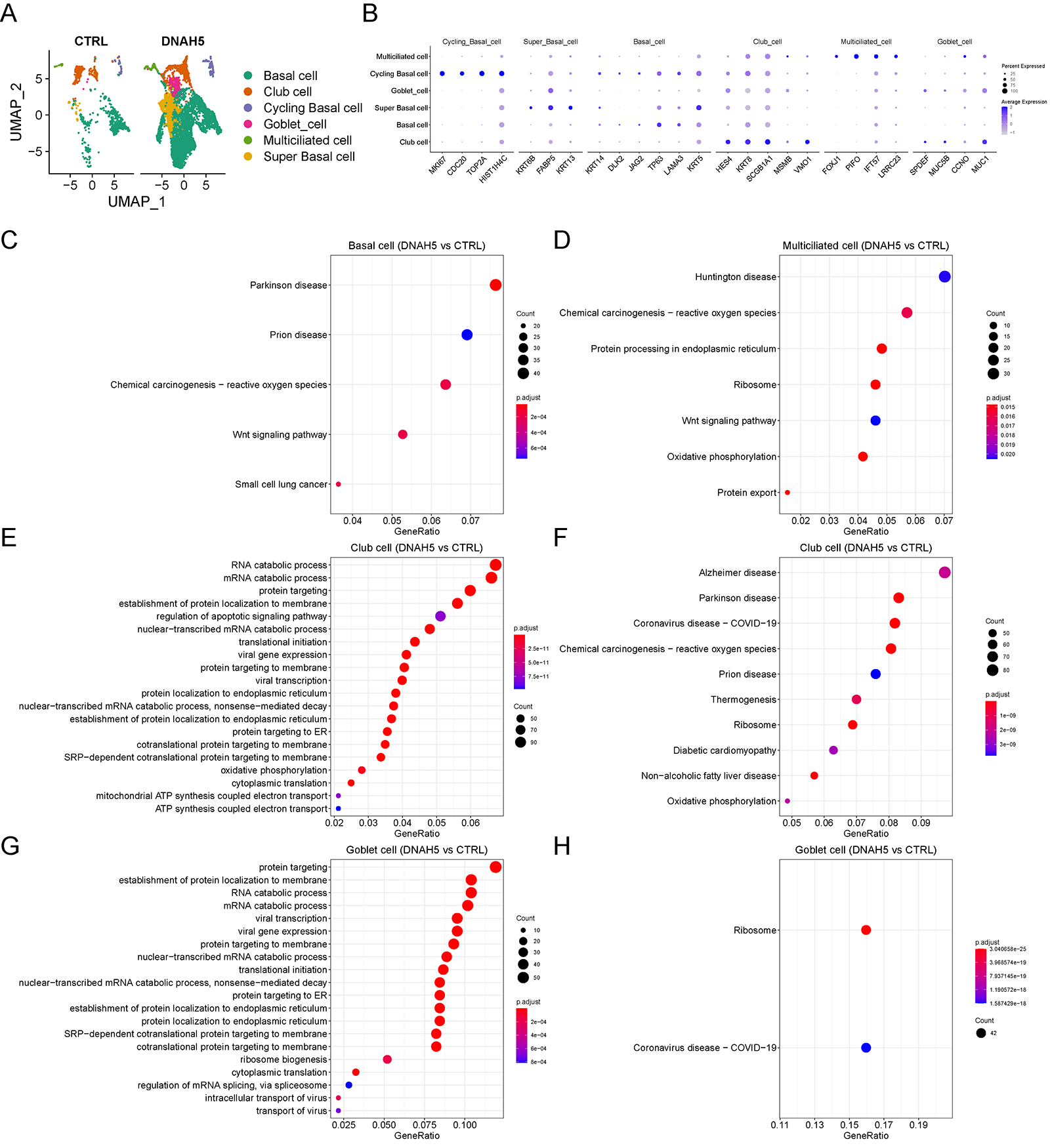

Supplement: Supplementary file 1 [file cells-11-04013-s001.zip › Figure S1.tif]
